# The Inhibition of Zinc Excitotoxicity and AMPK Phosphorylation by a Novel Zinc Chelator, 2G11, Ameliorates Neuronal Death Induced by Global Cerebral Ischemia

**DOI:** 10.3390/antiox11112192

**Published:** 2022-11-05

**Authors:** Dae Ki Hong, Jae-Won Eom, A Ra Kho, Song Hee Lee, Beom Seok Kang, Si Hyun Lee, Jae-Young Koh, Yang-Hee Kim, Bo Young Choi, Sang Won Suh

**Affiliations:** 1 Department of Physiology, College of Medicine, Hallym University, Chuncheon 24252, Korea; 2 Department of Integrative Bioscience and Biotechnology, Sejong University, Seoul 05006, Korea; 3 Neuroregeneration and Stem Cell Programs, Institute for Cell Engineering, Johns Hopkins University School of Medicine, Baltimore, MD 21205, USA; 4 Department of Neurology, Johns Hopkins University School of Medicine, Baltimore, MD 21205, USA; 5 Neural Injury Research Laboratory, Department of Neurology, University of Ulsan College of Medicine, Seoul 05505, Korea; 6 Department of Physical Education, Hallym University, Chuncheon 24252, Korea; 7 Institute of Sport Science, Hallym University, Chuncheon 24252, Korea

**Keywords:** global cerebral ischemia, zinc, AMP-activated protein kinase, 2G11, neuronal death

## Abstract

AMP-activated protein kinase (AMPK) is necessary for maintaining a positive energy balance and essential cellular processes such as glycolysis, gene transcription, glucose uptake, and several other biological functions. However, brain injury-induced energy and metabolic stressors, such as cerebral ischemia, increase AMPK phosphorylation. Phosphorylated AMPK contributes to excitotoxicity, oxidative, and metabolic problems. Furthermore, brain disease-induced release of zinc from synaptic vesicles contributes to neuronal damage via mechanisms including ROS production, apoptotic cell death, and DNA damage. For this reason, we hypothesized that regulating zinc accumulation and AMPK phosphorylation is critical for protection against global cerebral ischemia (GCI). Through virtual screening based on the structure of AMPK subunit alpha 2, we identified a novel compound, 2G11. In this study, we verified that 2G11 administration has neuroprotective effects via the blocking of zinc translocation and AMPK phosphorylation after GCI. As a result, we demonstrated that 2G11 protected hippocampal neurons against GCI and OGD/R-derived cellular damage. In conclusion, we propose that AMPK inhibition and zinc chelation by 2G11 may be a promising tool for preventing GCI-induced hippocampal neuronal death.

## 1. Introduction

Global cerebral ischemia (GCI), predominantly a prevalent cardiac arrest-induced rapid decrease in systemic blood flow toward to the brain, is characterized by an intensive loss of neurons in the hippocampus and cortex [1]. Early treatment and steps to prevent ischemic stroke-induced brain damage are important because severe neuronal loss and behavior impairment occur acutely following GCI. Although adequate treatments such as resuscitation or thrombolytic processes are conducted within an acute phase against ischemic conditions, additional brain damage occurs via blood reperfusion [2]. Prolonged brain ischemia activates pathological processes causing dysfunction of the blood–brain barrier, neuroinflammation, accumulation of phosphorylated tau, reactive oxygen species (ROS)-induced oxidative damage, and excitotoxicity [3,4,5,6]. Accumulated and overloaded zinc in the neurons from diverse neurological injuries that involve hypoglycemia [7], epilepsy [8], traumatic brain injury [9], and ischemic stroke [10] exacerbates additional brain damage.

Zinc is an abundant metal ion in the brain and is widely distributed in the central nervous system, especially the brain hippocampus [11]. In the brain, zinc is located in the intra-neuronal space, as well as in synaptic vesicles enveloped with an excitatory neurotransmitter, i.e., glutamate [12,13]. Zinc transportation-associated transporters, involving zinc transporters (ZnTs) and Zrt-, Irt-like proteins (ZIPs) located in the lysosome, Golgi complex, synaptic vesicles, and plasma membrane, regulate the zinc concentration within the intra- and extra-neuronal space [14]. Several studies have clarified the physiological or pathophysiological brain functions of zinc. An adequate concentration of zinc contributes to the promotion of hippocampal neurogenesis [15], zinc deficiency reduces DNA synthesis for cell differentiation [16], and a large amount of zinc released from pr-synaptic vesicles from several neurological disorders, particularly ischemic stroke, leads to pathological cell death cascades [17,18]. In particular, large amounts of released zinc from presynaptic vesicles has been shown to accumulate in the mitochondria, leading to neuronal excitotoxicity [19,20]. Consequently, mitochondrial membrane potential (ΔΨm) is reduced, with an increased production of reactive oxygen species (ROS) [17,20,21]. As mentioned above, zinc-induced neurotoxicity under neuropathological conditions causes deficiencies in energy production, which may upregulate AMP-activated protein kinase (AMPK) against metabolic stress [22].

AMPK is a hetero-trimeric complex that consists of α, β, and γ subunits. The α subunit of AMPK has catalytic activity, whereas β and γ have regulatory activity. AMPK signaling is activated by several physiological signals, including energy imbalance and metabolic stress [22,23]. The AMPK activation process is essential for glycolysis, gene transcription, protein synthesis, and cell growth under normal physiological conditions. However, several reports have verified that abnormal AMPK phosphorylation can contribute to pathophysiological regulation, which induces cell death cascades [24]. A previous study demonstrated that a high concentration of zinc (300 μM) treated in cultured cortical neurons revealed zinc-induced neurotoxicity, which increased apoptotic proteins, including cleaved caspase-3 and Bim. Moreover, the phosphorylation of AMPK is time-dependently increased with a high concentration of zinc-treated neurons [25]. Moreover, AMPK is highly activated during excitotoxic, oxidative damage, and metabolic stress, including a transient middle cerebral artery occlusion model of ischemic stroke and oxygen glucose deprivation/reperfusion conditions [26], Alzheimer’s disease [27], and synucleinopathies [28]. Although AMPK is essential for maintaining energy balance, the inhibition of ischemic brain injury-induced AMPK phosphorylation by using the AMPK inhibitor compound C (Cpd C) may contribute to stroke treatment [26,29]. In addition, AMPK alpha-2 knockout mice revealed a neuroprotective effect following ischemic stroke [29], but there were no significant differences between wild type and AMPK alpha-1 knockout mice. Collectively, the phosphorylated AMPK alpha-2 subunit could be detrimental to neurological disorders.

On the basis of the molecular structure of AMPKα2, a novel chemical compound, 2G11 (*N*-(2-((2-(1*H*-indol-3-yl)ethyl)amino)-2-oxoethyl)-3-phenylbenzo[c]isoxazole-5-carboxamide), was identified. In the previous report, we showed that 2G11 reduces zinc- or calcium-overloaded neuronal death, oxidative stress-mediated neurotoxicity, and staurosporine-induced apoptosis in mouse cerebrocortical cultures. In particular, focal cerebral ischemia from a permanent middle cerebral artery occlusion (MCAO)-induced brain infarct size was significantly decreased compared to a Cpd C-treated group [30].

Following the above demonstration of the novel compound, 2G11, we report that the regulation of overloaded zinc and phosphorylated AMPK in the brain by administering 2G11 during the acute and chronic periods protects against pathological brain damage from GCI. Because ischemic stroke-induced brain damage is complicated and not restricted to one cascade, we assert that multiple approaches to this target are effective and can more easily solve ischemic problems such as zinc overload and AMPK phosphorylation.

## 2. Materials and Methods

### 2.1. Experimental Animals

Animal care and handling were conducted according to the National Institutes of Health guidelines. Experimental procedures were approved by the Hallym University (Protocol # Hallym 2019-70) Institutional Animal Care and Use Committee of the College of Medicine. In the current study, male Sprague-Dawley (SD) rats (weight: 310–320 g; age: 8 weeks; DBL CO., Chungcheongbuk-do, Eumseong-gun, Republic of Korea) were used. All animals were housed in an animal care room that consistently maintained the temperature (22 ± 2 °C), humidity (55 ± 5%), and day and night light cycle (12 h interval).

### 2.2. Selection of Novel Zinc Chelator with Inhibition of AMPK Phosphorylation

Kim et al. proposed a zinc chelator based on binding activity to AMPK alpha-2 sites [31]. The selected 40 chemical compounds were analyzed for AMPK enzyme activity using an AMPK activity assay kit (CycLex, Nagano, Japan) and recombinant AMPK (α2/β1/γ1; CycLex, Japan) (Figure S1A). Moreover, high-dose zinc treatment (400 µM)-induced neurotoxicity was significantly decreased in compound #35 (Figure S1B). Consequently, compound #35 (2G11, *N*-(2-((2-(1*H*-indol-3-yl)ethyl)amino)-2-oxoethyl)-3-phenylbenzo[c]isoxazole-5-carboxamide) was adopted to be used in this study.

### 2.3. Primary Neuronal Culture

A pregnant female rat on embryonic day 18 (E18) was anesthetized with 2–3% isoflurane (ventilated using a mix gas of 70% nitrous oxide and 30% oxygen). The obtained E18 hippocampus was dissociated with TrypLE (without phenol red, Gibco, Denmark) and cultured with a neurobasal medium including B-27 (ThermoFisher, Waltham, MA, USA), GlutaMAX (Gibco, Denmark) supplemented with penicillin. Isolated hippocampal neurons were plated on sterilized coverslips coated with a poly-l-lysine solution (Sigma-Aldrich, St. Louis, MO, USA). Cells with a concentration of 5 × 10^5^ each were plated in a 24-well plate and cultured in a CO_2_ incubator (37.5 °C maintained). After the adequate maturation of cultured neurons for 2 weeks, neurons were fixed using 4% paraformaldehyde (PFA) for 15 min at room temperature. Fixed neurons were stored in a 4 °C incubator for immunostaining.

### 2.4. Oxygen–Glucose Deprivation/Reperfusion

On the last day of primary neuron maturation for 2 weeks, the neurobasal medium was changed to an oxygen–glucose deprivation (OGD) medium. The 24-well-plated neurons were placed in a hypoxia chamber, and N_2_ (95%) was flushed using a CO_2_ (5%) mixture gas for eliminating the air atmosphere for 10 min. Afterward, the hypoxia chamber was brought back to the incubator at 37 °C for the desired OGD exposure time. After the termination of OGD exposure, the OGD medium was removed, and the neurons were gently rinsed twice with Hanks’ balanced salt solution (HBSS). In order to induce cell damage caused by oxygen–glucose reperfusion, the medium from HBSS was exchanged with a neurobasal medium and incubated for 24 h. In the 2G11 treatment group, the reperfusion medium was additionally dissolved with 2G11 (20 µM). After reperfusion, the cultured neurons were fixed with 4% PFA and stored at 4 °C.

### 2.5. Cell Viability Assay

After 2 weeks of neuronal maturation, after subjection to the sham and OGD/R, the cultured primary neurons (concentration 3 × 10^4^ cells, placed on 96-well plate) were reacted with an EZ-Cytox solution (cell viability assay kit, DoGenbio CO., Korea) that was added to the neurons at a volume of 10 µL with 100 µL of plating medium. Absorbance was measured and calculated using a microplate reader (Spectramax, Molecular Devices CO., San Jose, CA, USA) at 450 nm.

### 2.6. Global Cerebral Ischemia Disease Modeling

Eight-week-old SD rats were used to demonstrate whether the novel compound, 2G11, has neuroprotection against global cerebral ischemia (GCI)-induced brain damage. Rats were anesthetized with 2–3% isoflurane, and ventilated using nitrous oxide (70%) and oxygen (30%). To avoid hypothermic conditions, a homeothermic monitoring heating pad (Harvard Apparatus, Holiston, MA, USA) was used, and body temperature was consistently checked during the disease modeling procedures. The right femoral artery was cannulated using a polyethylene tube for arterial blood pressure monitoring and transient blood drain for hypotension conditions. Bilateral common carotid arteries (CCAs) were separated from the vagus nerve using a surgical microscope (SZ61, Olympus, Shinjuku, Japan). An electroencephalogram (EEG) was consistently monitored using an electrode placed in bilateral burr holes. After the initiation of bilateral CCA clamping and blood withdrawal from the femoral artery (blood pressure range: systolic: 50 mmHg; diastolic: 40 mmHg), the EEG became isoelectric. After 7 min of isoelectricity, blocked blood circulation to the brain was restored by unclamping CCAs and blood reperfusion. Next, 2G11 (20 µg/kg, dissolved in 0.9% normal saline, once per day) was immediately injected for 3 and 28 days intraperitoneally after termination of the GCI surgery. Sham surgery was restricted to the incision of skin and dissection of the femoral artery/bilateral common carotid artery, without cannulation of femoral artery and occlusion of the bilateral common carotid arteries.

### 2.7. Tissue Preparation and Histological Staining

For deep anesthesia, rats were given urethane (1.5 g/kg, i.p.) dissolved in 0.9% NaCl. A toe pinch was used to check the effectiveness of the anesthesia. Anesthetized rats were transcardially perfused with 0.9% NaCl, followed by 4% paraformaldehyde (PFA) dissolved in phosphate-buffered saline (PBS). The obtained brain after perfusion was immersed in 4% PFA for post-fixation over 1 h. Next, the brain was transferred to 30% sucrose for cryoprotection over several days. Thereafter, the brain was coronally sectioned to 30 μm thickness using a cryostat microtome (CM1850, Leica, Germany).

To verify hippocampal neuronal death, brain slices from −2.40 to −4.8 mm relative to the bregma were placed on gelatin-coated slides (ThermoFisher Scientific, Waltham, MA, USA) and immersed in a 0.001% Fluoro-Jade B (FJB; Histo-Chem Inc., Jefferson, AR, USA) solution for 30 min and a 0.06% potassium permanganate solution for 15 min. The brain was stained with fluorescence FJB with an FITC filter in a wavelength range from 450 to 490 nm.

To detect Nissl bodies with granular structures in the neurons, slide-mounted brain sections were immersed in a 0.1% cresyl violet solution for 10 min, washed in running tap water, and dehydrated in 50%, 70%, 80%, and 90% ethanol, followed by rehydration in ethanol reverse and in a cresyl violet solution for 5 min and re-dehydration. These processes were terminated in xylene for clearing. The cresyl violet-stained brain was observed using a bright-field microscope.

### 2.8. Zinc Staining

The zinc-specific brain staining method using *N*-(6-methoxy-8-quinolyl)-*para*-toluene sulfonamide (TSQ) was previously explained [32,33]. Briefly, rats were sacrificed 3 days after GCI using 5% isoflurane for deep anesthesia. The brain was rapidly removed and then frozen in powdered dry ice. The brain was unfixed with 4% PFA and then coronally sectioned at 10 μm thickness using a −15 °C cryostat microtome. The sections were reacted with a 4.5 μM TSQ solution (Molecular Probes, Eugene, OR, USA) in 140 mM sodium barbital and sodium acetate (pH 10.5) for 60 s, and then washed for 60 s in 0.9% saline. The TSQ-positive fluorescence signal was captured using a fluorescence microscope (Olympus upright microscope, 360 nm UV light) and photographed using an INFINITY3-1 camera (Lumenera Co., Ottawa, Canada) with INFINITY software. The intensity of TSQ was measured using ImageJ and expressed as mean gray values.

### 2.9. Perfusion and Processing for Immunostaining

At the end of the experiment, all experimental animals were anesthetized with urethane (1.5 g/kg, i.p.) and transcardially perfused with 0.9% normal saline followed by 4% paraformaldehyde in a 0.1 M sodium phosphate buffer. Brain samples were removed, postfixed for 1 h, and stored in 30% sucrose for cryoprotection at 4 °C. Brain sections were washed three times in 0.01 M PBS after 1.2% hydrogen peroxide incubation to block endogenous peroxide activity. Primary neurons fixed with 4% PFA and the sectioned brains were incubated with phospho-AMPK (Abcam, 1:500), anti-MAP2 (Millipore, 1:500), anti-nNOS (ThermoFisher, 1:500), anti-4HNE (1:500), anti-nitrotyrosine (Abcam, 1:500), anti-phospho-TAU S396 (Abcam, 1:1000), anti-PSD95 (Invitrogen, 1:200), anti-IgG (Vector laboratories, 1:250), anti-SMI-71 (Covance, 1:500), anti-GFAP (Abcam, 1:1000), anti-AQP4 (Cell signaling, 1:500), anti-NeuN (Millipore, 1:500), anti-MMP9 (Abcam, 1:500), and anti-EGR1 (Cell signaling, 1:200) in a 4 °C incubator. Afterward, brain sections and cultured neurons were incubated with the fluorescence-conjugated secondary antibody for 2 h at room temperature.

### 2.10. Western Blot Analysis

Rat brain hippocampal samples were lysed in a lysis buffer containing a protease inhibitor (11697498001, Sigma, USA), a phosphatase inhibitor (4906845001, Sigma, USA), and an RIPA buffer (IBS-BR002, iNtRON, Republic of Korea), and then centrifuged for 20 min at 14,000 rpm, at 4°C. Supernatant protein quantification was carried out using a Bradford protein assay. Quantified supernatant was boiled in an SDS loading buffer. Separately divided proteins were transferred to a PVDF membrane after SDS-PAGE. Primary antibodies to the following target antigens were used: phospho-AMPK (Abcam, 1:1 k), AMPKα (Cell signaling, 1:1 k), MMP-9 (Abcam, 1:1 k), β-actin (Cell signaling, 1:10 k), HSP-70 (Enzo, 1:1 k), phospho-Tau (Abcam, 1:50 k), and PSD-95 (Invitrogen, 1:1 k).

### 2.11. Behavior Testing

***Modified neurological severity score****(mNSS).* The neurological function becomes dysfunctional after GCI. GCI-induced neurological dysfunction was measured using an mNSS test. Neurological function was graded from 0 to 18 (non-neurological dysfunction score: 0; maximum neurological deficits score: 18) [34]. Rats were tested using mNSS after 4 h, 1 day, 4 days, 7 days, 14 days, 21 days, and 28 days after GCI and the sham surgery. The criteria of mNSS are as follows: (1) flexion by raising tail (three points); (2) walking on floor (three points); (3) vision, tactile, and limb muscles (two points); (4) maintaining a balance on the beam (six points); (5) reflex absence and seizure activity (four points).

***Morris water maze****(MWM).* To test whether 2G11 treatment has neuroprotection against GCI-induced impairment of cognitive and memory function, rats were trained with an MWM test for five consecutive days initiated 3 weeks after GCI in a water-filled tub (120 cm diameter) with a platform (13 cm diameter) that submerged below the water surface (1 cm). The water tub was divided into four quadrants, and the escape platform was located in the middle of the target quadrant. Each subject underwent four trials/day, and the maximum finding hidden platform time was 120 s. After 5 days of task acquisition, their behavior was measured for 120 s without a platform as a probe trial. All trials were analyzed for escape latency, target crossing, distance to target, and time in target quadrant using Smart software [35] (Panlab, Harvard apparatus, Holliston, MA, USA). Regardless of whether rats reached or did not reach the platform within the acquisition time, they were returned back to the cage and dried to avoid hypothermia.

### 2.12. Quantification

Brain sections were collected from −2.40 to −4.8 mm relative to the bregma. Firstly, to count the number of immunoreactive and dye-stained tissues, brain sections were measured using Photoshop CS5. Loaded images were manually counted for positive and reactive cells (menu: Analyze/Count tool). Secondly, to measure immunofluorescence intensity, hippocampal regions from the brain sections were manually measured using Image J. Loaded images were changed to 8 bit (menu option: Image/Color/Split Channels), and the converted images were then measured by a mean gray value (menu option: Analyze/Measure). Thirdly, to measure hippocampal atrophy, i.e., lateral ventricle enlargement and hippocampus volume, brain sections were manually analyzed using ToupView software. Each calibrated and loaded image was measured for lateral ventricle and hippocampus volume size (unit: mm^2^).

### 2.13. Statistical Analysis

All experimental data are presented as the standard error of the mean (SEM). Repeated-measures analysis of variance (ANOVA) was conducted to assess differences in mNSS scores and escape latency over time among groups. Other comparisons between vehicle- and 2G11-treated groups were analyzed using a Mann–Whitney U test. In order to compare the four groups, entire experimental data were analyzed using the Kruskal–Wallis test with Bonferroni post-hoc analysis. A *p*-value of <0.05 indicates statistical significance. All data were analyzed using IBM SPSS statistics software.

## 3. Results

### 3.1. 2G11 Reduces Zinc Accumulation and AMPK Phosphorylation after Oxygen Glucose Deprivation/Reperfusion in Primary Cultured Neurons

**Figure 1:**
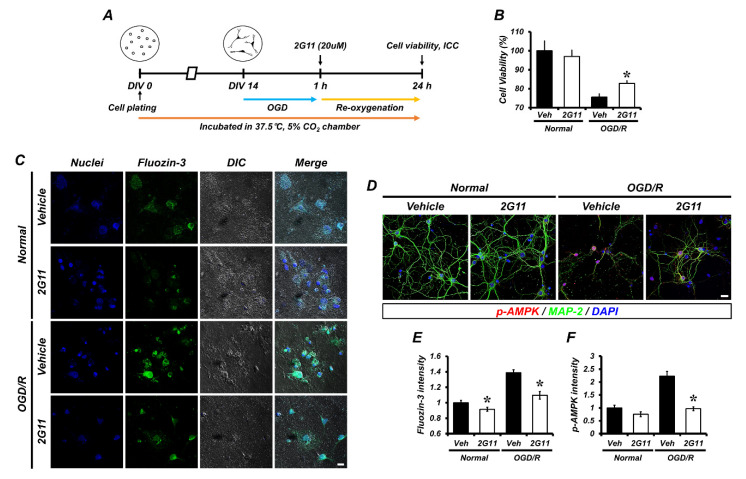
The 2G11 treatment protected cells against OGD/R injury in the primary hippocampal neuron culture. (**A**) Timeline showing the experimental design. (**B**) Cell viability was determined using the MTT assay. Data are the mean ± SEM. * *p* < 0.05 vs. vehicle-treated OGD/R group (Kruskal–Wallis test followed by a Bonferroni post-hoc test: chi square = 108.442, df = 3, *p* < 0.001). (**C**) Fluorescent and DIC merge micrographs of FluoZin-3 (green) expression in primary hippocampal neurons. Nuclei are stained with DAPI (blue). Scale bar = 10 μm. (**D**) Representative images of MAP-2 (green) and p-AMPK (red) co-staining in primary hippocampal neurons. Scale bar = 20 μm. (**E**) Bar graph showing the fluorescence intensity of FluoZin-3. Data are the mean ± SEM. * *p* < 0.05 vs. vehicle-treated OGD/R group (Kruskal–Wallis test followed by a Bonferroni post-hoc test: chi square = 79.532, df = 3, *p* < 0.001). (**F**) Bar graph showing the fluorescence intensity of p-AMPK. Data are the mean ± SEM. * *p* < 0.05 vs. vehicle-treated OGD/R group (Kruskal–Wallis test followed by a Bonferroni post-hoc test: chi square = 69.592, df = 3, *p* < 0.001).

Kim et al. proposed a zinc chelator based on the binding activity to AMPK alpha-2 sites [31]. Forty selected chemical compounds were analyzed for AMPK enzyme activity using an AMPK activity assay kit (CycLex, Japan) and recombinant AMPK (α2/β1/γ1; CycLex, Japan) (Figure S1A). Moreover, neurotoxicity induced by a high dose of zinc treatment (400 uM) was significantly decreased in Compound #35 (Figure S1B). Consequently, Compound #35 (2G11, *N*-(2-((2-(1*H*-indol-3-yl)ethyl)amino)-2-oxoethyl)-3-phenylbenzo[c]isoxazole-5-carboxamide) was adopted in this study. The selected novel chemical compound following virtual screening showed a binding affinity to chelatable zinc and inhibited AMPK phosphorylation (Figure S1E,F). To determine whether this chemical compound, 2G11, affects zinc translocation induced by oxygen glucose deprivation/reperfusion (OGD/R) and the phosphorylation of AMPK, we conducted immunocytochemistry staining on the primary cultured neurons (Figure 1D and Figure S1C). Strikingly, both zinc (Figure 1C) and phosphorylated AMPK (Figure 1D) were evidently increased in neurons treated with OGD/R + vehicle vs. OGD/R + 2G11, and the normal group treated with vehicle and 2G11 showed no differences in AMPK phosphorylation. However, zinc accumulation was significantly decreased in neurons by 2G11 treatment (Figure 1E,F). In addition, cell viability was reduced in the OGD/R + vehicle and was rescued by 2G11 treatment (Figure 1B).

### 3.2. 2G11 Attenuates Hippocampal Neuron Death, Zinc Accumulation, and AMPK Phosphorylation

**Figure 2:**
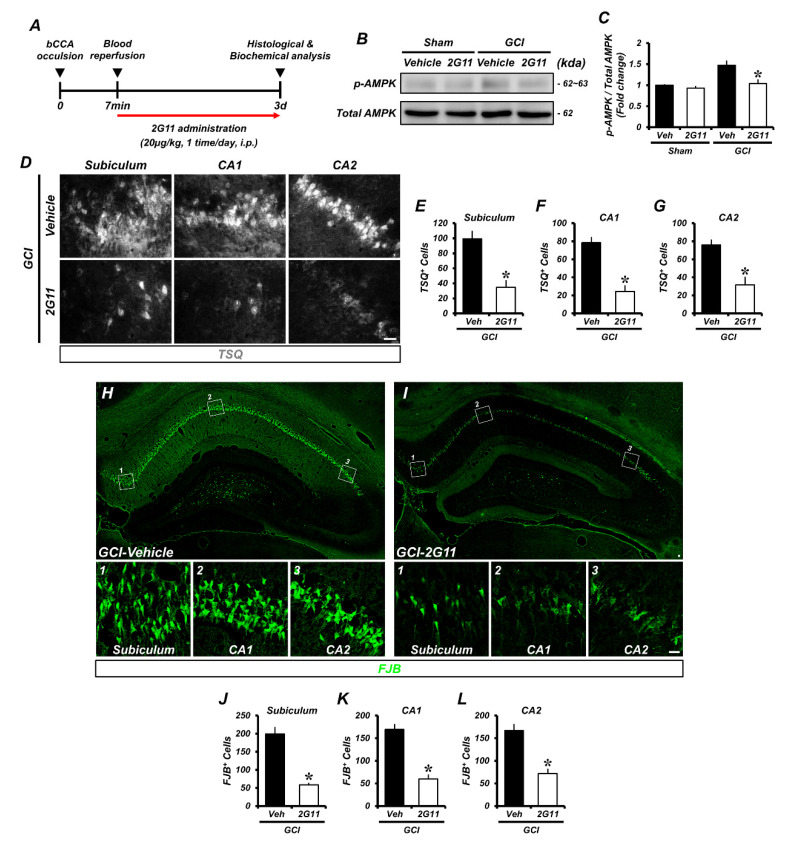
The 2G11 treatment reduced intracellular zinc accumulation, AMPK phosphorylation, and neuronal death after GCI. (**A**) Timeline displaying the experimental design. 2G11 was intraperitoneally administered once per day for 3 days before animal sacrifice. Rats were then killed 3 days after GCI. (**B**) Western blot analysis of p-AMPK (T183(α-1)/T172(α-2)) and total AMPK in the hippocampus after the sham surgery or GCI. (**C**) Western blot analysis of p-AMPK protein levels from the hippocampus. Data are the mean ± SEM (*n* = 3 from each sham group, *n* = 8 from each GCI group). * *p* < 0.05 vs. the vehicle-treated GCI group (Kruskal–Wallis test followed by a Bonferroni post-hoc test: chi square = 10.869, df = 3, *p* = 0.012). (**D**) Representative images showing sections of the hippocampal subiculum, CA1, and CA2 stained with TSQ to detect intracellular zinc accumulation. Scale bar = 25 μm. (**E**–**G**) Quantification of the number of TSQ^+^ cells from the hippocampal subiculum, CA1, and CA2 regions. Data are the mean ± SEM; *n* = 6 from each group, * *p* < 0.05 vs. the vehicle-treated GCI group (Mann–Whitney U test: *z* = 2.882, *p* = 0.002). (**H**,**I**) Representative images of degenerating neurons (FJB; green) in the subiculum, CA1, and CA2 from the hippocampus of the vehicle- and 2G11-treated groups after GCI. Scale bar = 20 μm. (**J**–**L**) Quantification of the number of FJB-positive cells from the hippocampal subiculum, CA1, and CA2 areas. Data are the mean ± SEM; *n* = 4 from each group, * *p* < 0.05 vs. the vehicle-treated GCI group (Mann–Whitney U test, subiculum: *z* = 2.309, *p* = 0.029; CA1: *z* = 2.309, *p* = 0.029; CA2: *z* = 2.309, *p* = 0.029).

To verify whether 2G11 (molecular structure, Figure S1D) regulates GCI-induced hippocampal neuron death, zinc translocation, and AMPK phosphorylation, we stained samples with Fluoro-Jade B (FJB), 6-methoxy-(8-*p*-toluenesulfonamido)quinoline (TSQ), and phospho-AMPK. The 2G11-treated group showed a strikingly lower number of FJB-positive cells in the hippocampal subiculum and in the cornu ammonis 1 (CA1) and CA2 regions compared to the vehicle-treated control group 3 days after GCI (Figure 2H–L). In addition, the phospho-AMPK-positive immunofluorescence signal was decreased in the 2G11-treated group compared to the vehicle-treated control group (Figure S2A–D). Immunoblotting showed GCI-induced AMPK phosphorylation. Vehicle- and 2G11-treated sham groups showed no differences in AMPK phosphorylation. However, the GCI + vehicle-treated group showed AMPK phosphorylation, which was downregulated by 2G11 treatment (Figure 2B,C). To determine whether 2G11 attenuates excessive zinc translocation to hippocampal neurons, we stained TSQ to verify vesicular zinc distribution. Excessively released zinc from synaptic vesicles in hippocampal regions was observed in the GCI-vehicle treated group. Zinc translocation following GCI was markedly reduced in the 2G11 treatment group (Figure 2D–G).

### 3.3. 2G11 Treatment Rescues Global Cerebral Ischemia-Induced Oxidative Damage

**Figure 3:**
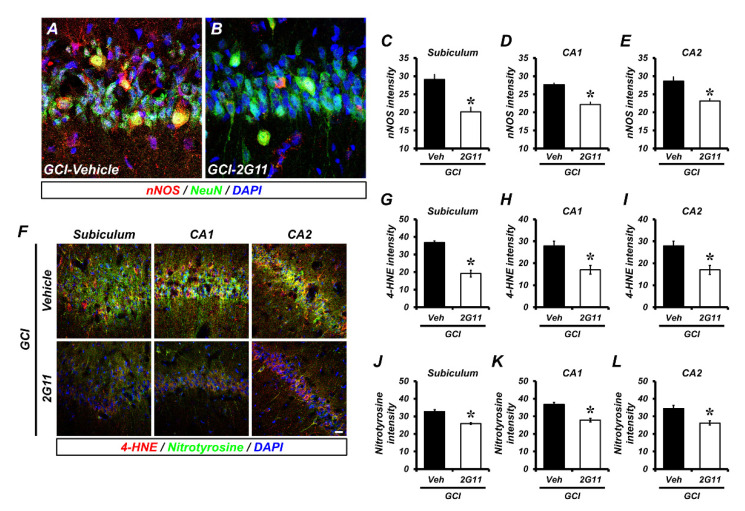
The 2G11 treatment reduced oxidative damage after GCI. (**A**,**B**) Representative images of NeuN (green) and nNOS (red) double immunostaining on the hippocampal CA1 from the vehicle- and 2G11-treated groups after GCI. Nuclei are stained with DAPI (blue). Scale bar = 10 µm. (**C**–**E**) Quantification of the immunofluorescence intensity of nNOS immunoreactivity as determined in the subiculum, CA1, and CA2 regions of the hippocampus. Data are the mean ± SEM; *n* = 4. * *p* < 0.05 vs. the vehicle-treated GCI group (Mann–Whitney U test, C: *z* = 2.309, *p* = 0.029; D: *z* = 2.309, *p* = 0.029; E: *z* = 2.309, *p* = 0.029). (**F**) Double-label confocal micrographs of nitrotyrosine (green) and 4-HNE (red) in the hippocampal subiculum, CA1, and CA2 for detecting lipid peroxidation and nitrosative stress, respectively. Nuclei are stained with DAPI (blue). Scale bar = 20 μm. (**G**–**L**) Bar graphs showing the fluorescence intensity of 4-HNE (**G**–**I**) and nitrotyrosine (**J**–**L**) immunoreactivity in the same hippocampal region after GCI. Data are the mean ± SEM; *n* = 5. * *p* < 0.05 vs. the vehicle-treated GCI group (Mann–Whitney U test, G: *z* = 2.611, *p* = 0.008; H: *z* = 2.611, *p* = 0.008; I: *z* = 2.611, *p* = 0.008; J: *z* = 2.842, *p* = 0.003; K: *z* = 2.842, *p* = 0.003; L: *z* = 2.517, *p* = 0.01).

One strategy to overcome ischemic brain damage is the regulation of reactive oxygen species (ROS) and reactive nitrogen species (RNS) production. Excessively released zinc from synaptic vesicles after GCI led to increased nNOS expression and activity in hippocampal neurons. nNOS-positive fluorescence was significantly decreased in the 2G11-treated group against GCI (Figure 3A–E). The product of lipid peroxidation by reactive oxygen species was evaluated using 4-HNE, and tyrosine nitration by reactive nitrogen species was evaluated by nitrotyrosine staining. Using immunofluorescence analysis, 4-HNE was extensively found to co-localized with nitrotyrosine in the hippocampal subiculum and in the CA1 and CA2 regions after GCI and strikingly decreased after 2G11 treatment (Figure 3F–L). Subsequently, oxidative damage-triggered microtubule damage in the hippocampus was ameliorated by 2G11 treatment after GCI (Figure S3A–E).

### 3.4. Global Cerebral Ischemia-Induced Tau Phosphorylation and Synaptic Damage Prevented by 2G11 Treatment

**Figure 4:**
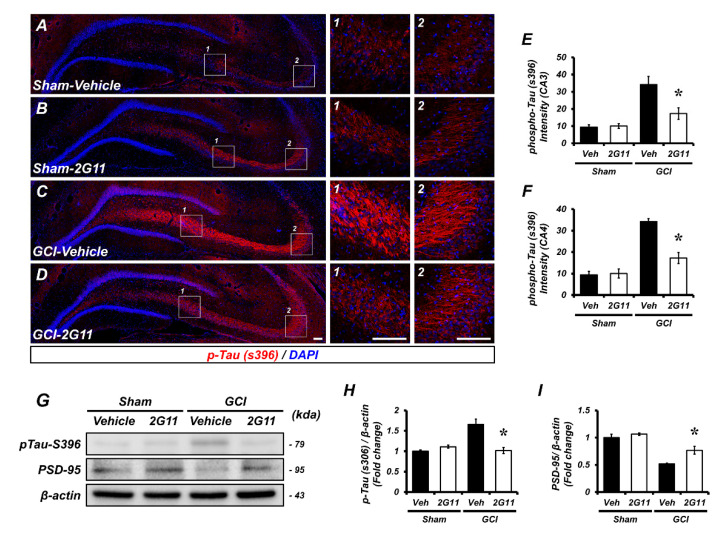
The 2G11 treatment restored GCI-induced phosphorylation of tau and loss of synaptic proteins. (**A**–**D**) Representative images of p-tau (S396; red) in the hippocampal mossy fiber from the vehicle- and 2G11-treated sham groups and the vehicle- and 2G11-treated GCI group 3 days after the sham surgery or GCI. Nuclei stained with DAPI (blue). Scale bar = 100 μm. (**E**,**F**) Quantification showing the immunofluorescence intensity of phospho-tau (serine 396) 3 days after GCI**.** Data are the mean ± SEM; *n* = 4–5. * *p* < 0.05 vs. the vehicle-treated GCI group (Kruskal–Wallis test followed by a Bonferroni post-hoc test; E: chi square = 9.165, df = 3, *p* = 0.027; F: chi square = 9.412, df = 3, *p* = 0.024). (**G**–**I**) Western blot analysis of phospho-tau and PSD-95 in the hippocampus after the sham surgery or GCI. Quantification of phospho-tau and PSD-95 protein levels. Data are the mean ± SEM; *n* = 3–4. * *p* < 0.05 vs. the vehicle-treated GCI group (Kruskal–Wallis test followed by a Bonferroni post-hoc test; K: chi square = 9.379, df = 3, *p* = 0.025; L: chi square = 11.512, df = 3, *p* = 0.009).

Global cerebral ischemic brain damage triggers disruption of the neural structure involving the microtubule and tau. We sought to determine whether GCI-induced microtubule damage, by increasing the phosphorylation of tau, and synaptic loss was prevented by 2G11 treatment. Little phosphorylated tau (serine 396) was observed in the brain hippocampus, especially the CA3 and CA4 (mossy fiber) region, in the sham-operated groups. However, GCI triggers extensive tau phosphorylation and was reduced by 2G11 treatment (Figure 4A–D). Pre- and post-synaptic markers, synaptophysin, and PSD-95 immunoreactive fluorescence were highly observed in primary cultured neurons. OGD-induced synaptic damage was restored by 2G11 treatment (Figure S4). Western blotting analysis confirmed that GCI contributes to tau phosphorylation and synaptic loss, and it was preserved by 2G11 treatment (Figure 4G–I).

### 3.5. 2G11 Treatment Protects against Blood–Brain Barrier Damage after Global Cerebral Ischemia

**Figure 5:**
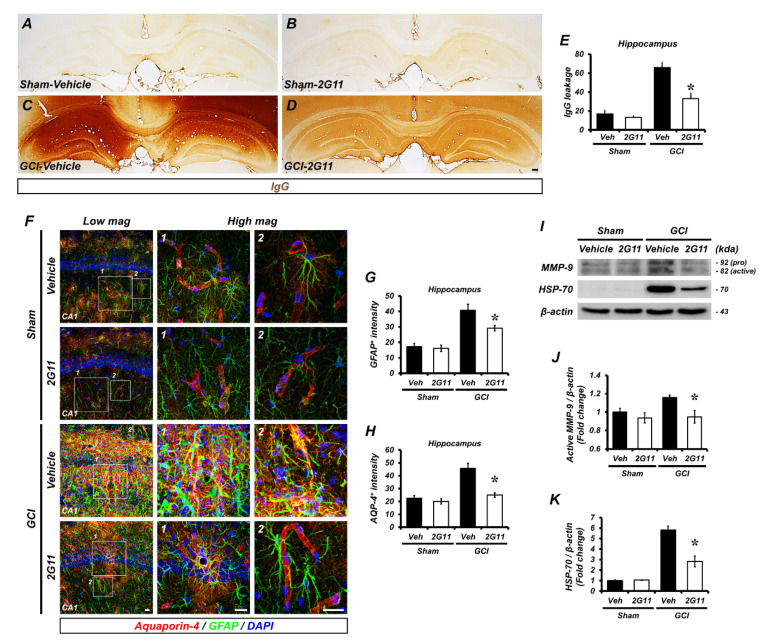
The 2G11 treatment reduced BBB disruption and MMP-9 activation after GCI. (**A**–**D**) Photomicrographs showing sections of the hippocampus stained for IgG from the vehicle-treated sham, 2G11-treated sham, vehicle-treated GCI, and 2G11-treated GCI groups 3 days after the sham surgery or GCI. Scale bar = 100 μm. (**E**) Graph representing IgG leakage from the hippocampus in rats treated with the vehicle or 2G11 3 days after the sham surgery or GCI. Data are the mean ± SEM; *n* = 6. * *p* < 0.05 vs. the vehicle-treated GCI group (Kruskal–Wallis test followed by a Bonferroni post-hoc test; chi square = 14.184, df = 3, *p* = 0.003). (**F**) Representative images showing BBB marker SMI-71^+^ endothelial protein co-labeled with the reactive astrocyte marker GFAP and the water channel protein marker AQP4 in the hippocampal CA1 from vehicle- and 2G11-treated rats 3 days after the sham surgery or GCI. Scale bar = 20 μm. (**G**,**H**) Bar graphs showing the immunofluorescence intensity of GFAP and AQP4 3 days after GCI**.** Data are the mean ± SEM; *n* = 5. * *p* < 0.05 vs. the vehicle-treated GCI group (Kruskal–Wallis test followed by a Bonferroni post-hoc test; G: chi square = 12.156, df = 3, *p* = 0.007; H: chi square = 13.091, df = 3, *p* = 0.004). (**I**) Western blot analysis of MMP-9 in the hippocampus after the sham surgery or GCI. Quantification of MMP-9 and HSP-70 protein levels from the hippocampus. Data are the mean ± SEM; *n* = 4. * *p* < 0.05 vs. the vehicle-treated GCI group (Kruskal–Wallis test followed by a Bonferroni post-hoc test: (**J**) chi square = 7.832, df = 3, *p* = 0.05; (**K**) chi square = 12.217, df = 3, *p* = 0.007).

We investigated whether 2G11 would protect or rescue destabilized blood–brain barrier (BBB)-induced water and immunoglobulin extravasation from GCI. We performed immunostaining that visualized immunoglobulin G leakage through the destroyed BBB in the vehicle- and 2G11-treated sham and GCI-operated groups (Figure 5A–D and Figure S5A). The immunoglobulin G (IgG)-positive signal showed that IgG leakage from a damaged BBB was extensive in GCI. However, the IgG-positive leakage was decreased in the 2G11-treated group compared with the GCI-vehicle group (Figure 5E). Moreover, we sought to determine whether ischemic stroke-induced brain edema, which is the dysregulated movement of fluid through the water channel aquaporin 4 (AQP4), can be regulated by 2G11 treatment. We conducted co-immunofluorescence staining in the hippocampal CA1 region (Figure 5F). The vehicle-treated GCI group displayed highly activated astrocytes, as well as disturbed blood vessels and aquaporin; conversely, 2G11 treatment reduced astrocyte activation and AQP-4 expression (Figure 5G,H). Western blotting analysis showed that GCI highly contributes to the expression of matrix metalloproteinase 9 (MMP-9) and heat-shock protein (HSP-70) and was downregulated by 2G11 (Figure 5I–K). In addition, we found that MMP-9 and HSP-70 were localized in the hippocampal CA1, whereas they were extensively expressed after GCI and reduced after 2G11 treatment (Figure S5B–D).

### 3.6. Chronic 2G11 Treatment Rescues Global Cerebral Ischemia-Induced Neurological Deficits, as well as Memory and Cognitive Impairments

**Figure 6:**
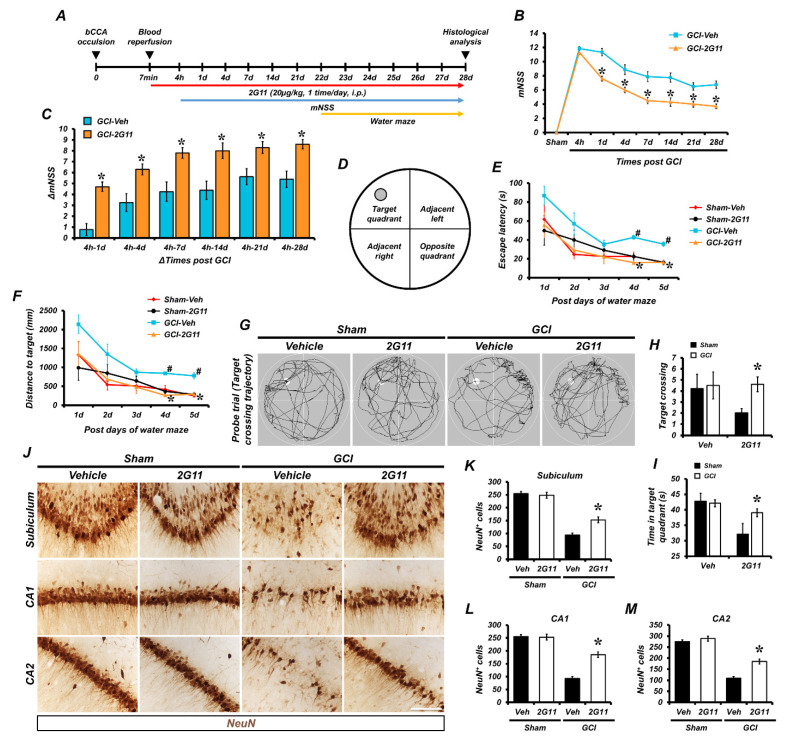
Neurologic impairment and cognitive decline following GCI were protected by 2G11 treatment. (**A**) Timeline displaying the experimental design. 2G11 was intraperitoneally administered once per day for 28 days before animal sacrifice. Rats were then sacrificed 28 days after GCI. (**B**) The mNSS determined in rats at 4 h, 1 day, 4 days, 7 days, 14 days, 21 days, and 28 days after GCI. mNSS score range from 0 to 18. A score of ‘0’ means that all tasks were completed, while a score of ‘18’ means that all tasks were failed. (**C**) Changes in mNSS score (ΔmNSS) were evaluated at various time intervals between the 4 h time point and the multiple predetermined time points thereafter. Data are the mean ± SEM; *n* = 7–10. * *p* < 0.05 vs. the vehicle-treated GCI group. (**D**) Schematic diagram of the tank for MWM performance. (**E**,**F**) Escape latency and distance to the target of the acquisition trial for five consecutive days starting on post-injury day (PID) 22. Data are the mean ± SEM; *n* = 7–10. * *p* < 0.05 vs. the vehicle-treated GCI group; ^#^ *p* < 0.05 vs. the vehicle-treated sham group. (**G**) Representative tracing image of swimming during the MWM probe trial. The target is indicated with a white circle. (**H**) Counts of target crossing during the probe trial. (**I**) Time spent in the target quadrant during the probe trial. Data are the mean ± SEM; *n* = 7–10. * *p* < 0.05 vs. the vehicle-treated GCI group (Kruskal–Wallis test followed by a Bonferroni post-hoc test: H: chi square = 25.886, df = 3, *p* < 0.05; I: chi square = 19.927, df = 3, *p* < 0.05). (**J**) Representative images show neuronal survival stained with NeuN 4 weeks after GCI. Scale bar = 100 μm. (**K**–**M**) The counts of NeuN-positive neurons in the hippocampal subiculum, CA1, and CA2. Data are the mean ± SEM; *n* = 6. * *p* < 0.05 vs. the vehicle-treated GCI group (Kruskal–Wallis test followed by a Bonferroni post-hoc test: K: chi square = 19.213, df = 3, *p* < 0.05; L: chi square = 19.467, df = 3, *p* < 0.05; M: chi square = 20.567, df = 3, *p* < 0.05).

We sought to determine whether GCI induced dysfunction in behavioral outcomes and whether 2G11 could rescue it. We considered a modified neurological severity score (mNSS) and conducted the Morris water maze (MWM) test. Neurological deficits were evaluated at predetermined time points following GCI, and 2G11 treatments resulted in a rescued behavior outcome, as shown by the decrease in the mNSS (Figure 6B) and the increase in ΔmNSS (Figure 6C), compared to the GCI-vehicle treated group. The mNSS result revealed that memory and cognitive functions were substantially impaired in the GCI-subjected group, and the group showed severe behavior problems with sensory, motor, reflex, and balance functions. Marked behavior recovery was observed in the 2G11-treated group after GCI in comparison with the vehicle group through the beam balance test (Videos 1–4). The MWM assay showed that cognitive and memory functions were substantially impaired in the GCI-subjected group. The subjects required a high amount of time to find a hidden platform submerged underwater and swam longer distances to the target from each quadrant during the five consecutive days 3 weeks after GCI. The 2G11-treated group displayed significantly reduced escape latency to the target (Figure 6E) and a reduced total distance to the target (Figure 6F) at 4 and 5 days during the MWM period. On the last day of MWM, target crossing and time in the quadrants of the probe trial were measured. The 2G11-treated group after GCI showed a significantly increased target crossing count in the probe trial and a higher amount of time spent in the target quadrant vs. the GCI-vehicle treated group (Figure 6H,I). Lastly, to determine whether the chronic period treatment of 2G11 has a protective effect against GCI, neuronal nucleus (NeuN) and cresyl violet staining was performed (Figure 6J and Figure S6A). A loss of NeuN-positive cells was found in the hippocampal regions after GCI, and they were restored by 2G11 treatment, but this was not significant in the sham-operated groups (Figure 6K–M). In addition, to determine whether the chronic treatment of 2G11 prevents GCI-induced brain atrophy from causing lateral ventricle enlargement and hippocampus shrinkage, we performed cresyl violet staining to measure lateral ventricle and hippocampus volume. Strikingly, 2G11 significantly reduced brain atrophy due to GCI (Figure S6A–C). Moreover, chronic 2G11 treatment showed an enhancement in synaptic plasticity and neuronal activity according to early growth response protein 1 (EGR-1) staining (Figure S6D). EGR-1-positive cells were observed in the brain hippocampus, and 2G11 treatment protected against the impairment of synaptic plasticity and neural activity after GCI (Figure S6E–G).

## 4. Discussion

The present study demonstrated that the modulation of phosphorylated AMP-activated protein kinase (AMPK) and excessively released zinc from synaptic vesicles using a novel compound 2G11 had neuroprotective effects against global cerebral ischemic brain injury. It is known that AMPK phosphorylation is triggered by metabolic stress, such as ischemic or hypoxic conditions [22,36,37]. One of the major deleterious neuron death cascades after brain ischemia is the zinc excessively released from synaptic vesicles, which induces several degenerating processes involving excitotoxicity, apoptosis, and necrosis [10,38,39,40].

We developed a novel compound, 2G11, to compensate for Cpd C limitations. This compound has a zinc chelation effect in extracellular zinc overload conditions [30]. Thus, the downregulation of AMPK phosphorylation and zinc overload in neurons after stroke may have therapeutic potential. Therefore, in the present study, we focused on both zinc overload and AMPK phosphorylation to prevent stroke-induced neuron death. Relationships between zinc and AMPK have not been well identified. Notably, several studies have shown that GCI-induced hippocampal neuron death and cognitive impairments were induced by AMPK phosphorylation and zinc translocation [7,26,41,42]. To test whether 2G11 has neuroprotective effects through the inhibition of AMPK phosphorylation and zinc chelation, we employed a GCI rat model.

Compound 2G11 was selected through serial structure-based virtual screening according to the binding activity of an AMPK-active site, and it showed neuroprotective effects against focal ischemic brain damage [30]. Accordingly, we applied 2G11 to GCI and then evaluated its role in several oxidative stress-related neuron death cascades, such as the activation of nitro oxide synthase (NOS), lipid peroxidation, and tyrosine nitration [40,43,44].

Our lab reported that excessive vesicular zinc released from presynaptic terminals and subsequent zinc accumulation into post-synaptic neurons induced multiple neuron death cascades, such as reactive oxygen species (ROS) production and poly(ADP-ribose) polymerase-1 (PARP-1) activation [45,46]. We also demonstrated that microglia activation and MMP-9 activation induced BBB disruption, which is also involved in zinc toxicity in the brain [41,47,48]. Cytoplasmic zinc increases were prevented by the intracerebroventricular (i.c.v) injection of the zinc chelator, CaEDTA [7,10]. Without zinc chelation, this intraneuronal zinc accumulation continued to increase for 24 h after GCI and hypoglycemia [49]. CaEDTA treatment prevented this continuous intracellular zinc accumulation when evaluated 24 h later, suggesting that zinc released from the synaptic vesicles translocated into the postsynaptic neurons several hours after GCI or hypoglycemia. We also reported that the inhibition of zinc influx through zinc transporters such as transient receptor potential melastatins (TRPMs) decreased hippocampal neuron death after GCI [42,50,51]. From these findings, we speculate that zinc release and translocation are key upstream steps in the sequence of events leading to neuronal death after GCI or hypoglycemia [7,46].

A neural network link with a complicated structure of synapses establishes brain connections [52]. Within the synaptic cleft, numerous neurotransmitters and neuromodulators involving glutamate, acetylcholine, and zinc are moved or engage in reuptake through several channels and receptors located in each synapse. Several brain disease-induced synaptopathies with dissociated synaptic connections have been observed in the brain, especially the hippocampal mossy fiber region [53,54]. Notably, we found that highly phosphorylated tau (serin 396) in mossy fiber was reduced by 2G11 administration. In general, tau protein was attached to neurons and stabilized the neuronal structure, indicating that it was disrupted by phosphorylation [55]. Moreover, the synaptic marker for the postsynaptic density protein-95 (PSD-95) fluorescence signal was decreased after GCI, which indicates impaired synaptic function. Synaptic damage during the acute phase following GCI may contribute to delayed neuronal loss, sensorimotor deficit, and cognitive impairment in the chronic phase [52,56]. Chronic 2G11 treatment recovers the loss of memory function, leading to motor function outcomes without toxicity.

GCI also disrupted blood–brain barrier (BBB) integrity as seen in focal ischemia, hypoglycemia, head trauma, and multiple sclerosis. When BBB breakdown occurs, the activation of non-neuronal cells, microglia, and the astrocytes and extravasation of brain substrates such as immunoglobulin are induced [15,57,58]. Ischemic stroke-induced BBB damage also increases the activation of matrix metalloproteinase-9 (MMP-9) [59,60], which is associated with tight junction components, resulting in a disruption of BBB integrity [61]. Thus, we tested whether 2G11 can reduce BBB disruption and the subsequent brain edema due to the activation of MMP-9 after GCI. We found that GCI-induced cerebral morphologic alteration, such as lateral ventricle enlargement and hippocampal atrophy, was significantly decreased by 2G11 administration, even at the chronic stage. When we performed the pharmacokinetic analysis of 2G11, we found that 2G11 could not cut across the BBB (data are not shown). We believe the 2G11 enters into the central nervous system via the damaged BBB after GCI.

We next focused on behavior outcomes after the long-term chronic administration of 2G11. On the basis of the neuroprotective effects of 2G11, we tested whether 2G11 administration showed any side-effects or restoration of cognitive impairments following GCI using several behavior assessment tests. Behavior disorders following stroke include neurological deficits, cognitive impairment, and memory dysfunction [62]. The present study demonstrated that long-term 2G11 treatment rescues neurologic deficits and memory function, according to an mNSS grade test and a water maze test, respectively. Furthermore, to verify that GCI-induced delayed neuronal loss was also rescued by the administration of 2G11, we evaluated hippocampal neuronal loss through neuronal nuclei (NeuN) staining 28 days after GCI. Long-term 2G11 administration also had no side-effects and reduced delayed neuronal loss, even after a prolonged ischemic period.

**Figure 7:**
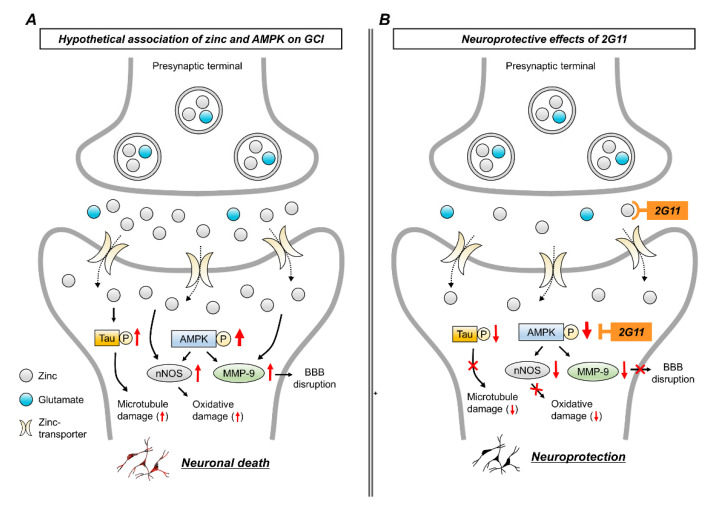
Proposed cascades of 2G11 effects on GCI-induced neuronal death. (**A**) Schematic illustration describing the possible cascades through which GCI induces neuronal damage via zinc overload and AMPK phosphorylation. (**B**) Neuroprotective effects of 2G11 on GCI: 2G11 chelates zinc and reduces AMPK phosphorylation, thus preventing hippocampal neuronal death after GCI.

Zinc and AMPK are important in maintaining physiological homeostasis, such as energy balance, protein synthesis, cell growth, and axonal and synaptic transmission [23,63]. However, several brain diseases trigger AMPK phosphorylation and release excessive zinc from the synaptic vesicles, leading to brain damage via several neuron death cascades (Figure 7A). The present study revealed that the inhibition of AMPK phosphorylation and zinc overload by 2G11 decreased GCI-induced zinc accumulation, neuronal death, microtubule disruption, BBB leakage, and cognitive impairment (Figure 7B). Collectively, the present results suggest that 2G11 may have therapeutic potential in treating GCI.

## 5. Conclusions

The present study suggests that the dual targeting of zinc and AMPK by 2G11 has therapeutic effects for preventing global cerebral ischemia-induced hippocampal neuron death.

## Data Availability

The datasets generated and/or analyzed during the current study are available from the corresponding authors on reasonable request.

## Supplementary Materials

The supporting information can be downloaded at https://www.mdpi.com/article/10.3390/antiox11112192/s1. Ref. [64] is mentioned in the Supplementary Materials.
