# Supplementary material for: The Inhibition of Zinc Excitotoxicity and AMPK Phosphorylation by a Novel Zinc Chelator, 2G11, Ameliorates Neuronal Death Induced by Global Cerebral Ischemia
Source: Antioxidants (Basel). 2022 Nov 5;11(11):2192. doi: 10.3390/antiox11112192 (PMC9686920; doi:10.3390/antiox11112192)
Supplement: Supplementary file 1 [file antioxidants-11-02192-s001.zip › antioxidants-1959523-supplementary.pdf]

Supplementary information for Antioxidants

The Inhibition of Zinc Excitotoxicity and AMPK Phosphorylation by a Novel Zinc Chelator, 2G11, Ameliorates Neuronal Death Induced by Global Cerebral Ischemia

Dae Ki Hong [1], Jae-Won Eom [2], A Ra Kho [3,4], Song Hee Lee [1], Beom Seok Kang [1], Si Hyun Lee [1], Jae-Young Koh [5], Yang-Hee Kim [2], Bo Young Choi [6,7,*] and Sang Won Suh [1,*]

1 Department of Physiology, Hallym University, College of Medicine, Chuncheon 24252, Korea
2 Department of Integrative Bioscience and Biotechnology, Sejong University, Seoul 05006, Korea
3 Neuroregeneration and Stem Cell Programs, Institute for Cell Engineering, Johns Hopkins University School of Medicine, Baltimore, MD 21205, USA
4 Department of Neurology, Johns Hopkins University School of Medicine, Baltimore, MD 21205, USA
5 Neural Injury Research Laboratory, Department of Neurology, University of Ulsan College of Medicine, Seoul 05505, Korea
6 Department of Physical Education, Hallym University, Chuncheon 24252, Korea
7 Institute of Sport Science, Hallym University, Chuncheon 24252, Korea
* Correspondence: bychoi@hallym.ac.kr (B.Y.C.); swsuh@hallym.ac.kr (S.W.S.)

This file includes:

Figure S1. Screening for novel chemical compound

Figure S2. 2G11 treatment reduces GCI-induced phosphorylation of AMPK

Figure S3. 2G11 treatment rescues GCI-induced microtubule damage

Figure S4. OGD/R-induced loss of synaptic proteins prevented by 2G11

Figure S5. 2G11 treatment preserves BBB disruption and heat shock protein expression

Figure S6. Chronic 2G11 treatment rescues GCI-induced brain atrophy, loss of synaptic plasticity and neuronal activity

Table S1. Modified neurological severity score points

Video S1. Behaviors of vehicle-treated sham group

Video S2. Behaviors of 2G11-treated sham group

Video S3. Behaviors of vehicle-treated GCI group

Video S4. Behaviors of 2G11-treated GCI group

A

Compound ID	Mean (% remaining activity)	SEM
#1	-1.2626	1.768
#2	-1.7934	0.072
#3	-0.9684	0.036
#4	5.5556	0.265
#5	4.2328	0.529
#6	2.381	0.794
#7	3.4392	0.265
#8	-0.2152	0.215
#9	0.5739	0.359
#10	-0.3945	0.179
#11	5.291	0.529
#12	-1.3245	0.662
#13	-0.9684	0.681
#14	-0.1435	0
#15	5.0265	0.265
#16	6.3492	0.529
#17	2.9101	0.265
#18	2.9053	0.681
#19	4.34	1.614
#20	-1.7575	0.036
#21	0.8967	0.251

Compound ID	Mean (% remaining activity)	SEM
#22	-0.5291	0
#23	1.0582	0
#24	5.0505	5.051
#25	-0.1793	0.538
#26	-1.2554	0.036
#27	5.8201	0
#28	3.9683	1.852
#29	0	0
#30	1	0.667
#31	0.3641	0.85
#32	0.1257	0.126
#33	2.9801	2.98
#34	0.3143	0.566
#35	0.1214	0.121
#36	0.2514	0.126
#37	7.3333	1
#38	5.6667	4.667
#39	1.667	3.333
#40	3.6667	0
**Compound C**	5.3	0.76

B

Compound ID	Significance
#1	0.5487
#2	0.8483
#3	0.5597
#4	0.0283
#5	0.9326
#6	0.0278
#7	0.0003
#8	0.0001
#9	0.8649
#10	0.1253
#11	0.0311
#12	0
#13	0.4384
#14	0.022
#15	0.2378
#16	0.2488
#17	0.0048
#18	0.3961
#19	0.759
#20	0.8889
#21	0.6141

Compound ID	Significance
#22	0.02
#23	0.8757
#24	0.8699
#25	0.487
#26	0.6624
#27	0.303
#28	0.0008
#29	0.1137
#30	0.2142
#31	0.1777
#32	0.7276
#33	0.2386
#34	0.1043
#35	0.0016
#36	0.0381
#37	0.029
#38	0.3225
#39	0.0027
#40	0.039
**Compound C**	0.0003

C

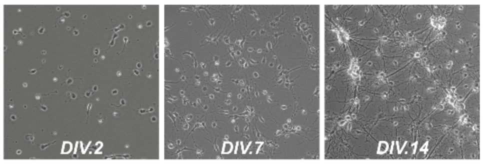

D

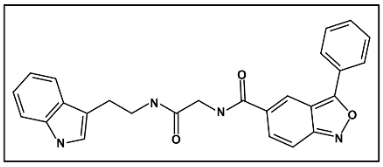

E

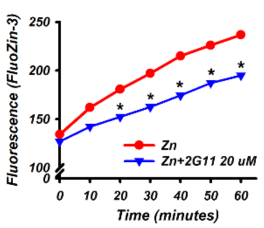

F

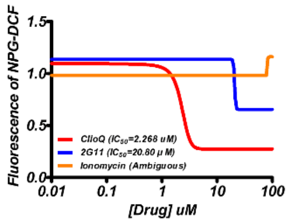

Figure S1. (**A**) Based on the molecular structure of the AMPK inhibitor Compound C (Cpd C), 40 compounds were selected to analyze AMPK enzyme activity. Compound #1 to #40 showed similar or better inhibitory effects of AMPK activity compared to Cpd C. (**B**) Zinc-induced neurotoxicity was analyzed using selected compounds in mouse primary cortical neurons. Cultured neurons were reacted with zinc (400 μm) for 10 min, and comparison and analysis were conducted for each compound. Compound #35 (2G11) has outstandingly neuroprotective effects from zinc-induced neurotoxicity. (**C**) Representative images showed that the consequent development of primary cultured neurons in this study. (**D**) The chemical structure of 2G11. (**E**) Intracellular zinc level was measured at the indicated time points after 15 min of exposure to 300 uM zinc with or without 20 uM 2G11 in primary mouse neuronal cultures. Values are mean ± SEM (n = 3). *$p < 0.05$ vs. zinc alone at the same time point (unpaired Student's t-test). (**F**) Zinc binding affinity was analyzed in the test tubes. Fluorescence of

Newport Green DCF (NPG-DCF) was measured after a 30 min reaction of zinc (20 uM) and the drugs (2G11, clioquinol, or ionomycin) at different doses (0, 0.2, 0.6, 2, 10, 20, 40, or 100 uM). Clioquinol and ionomycin were used as positive and negative controls, respectively. The half maximal inhibitory concentration (IC50) was calculated under Prism 5.

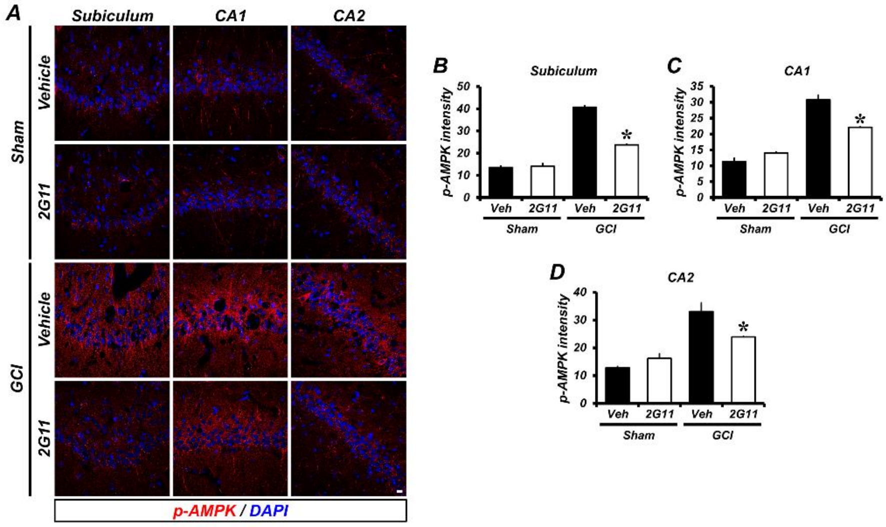

Figure S2. 2G11 treatment reduces GCI-induced phosphorylation of AMPK. (**A**) Immunofluorescent images representing the phospho-AMPK stained for anti-p-AMPK (T183(α-1)/T172(α-2), red) in the hippocampal subiculum, CA1, and CA2. Nuclei were counterstained with DAPI (blue). Scale bar = 10 μm. (**B-D**) Quantification of the immunofluorescence intensity of phospho-AMPK as determined in the hippocampal subiculum, CA1, and CA2 regions. Data are mean ± SEM; n = 3. *$p < 0.05$ vs. the vehicle-treated GCI group (Kruskal–Wallis test followed by a Bonferroni post-hoc test; B: Chi square = 9.359, df = 3, $p = 0.025$; C: Chi square = 9.974, df = 3, $p = 0.019$; D: Chi square = 10.385, df = 3, $p = 0.016$).

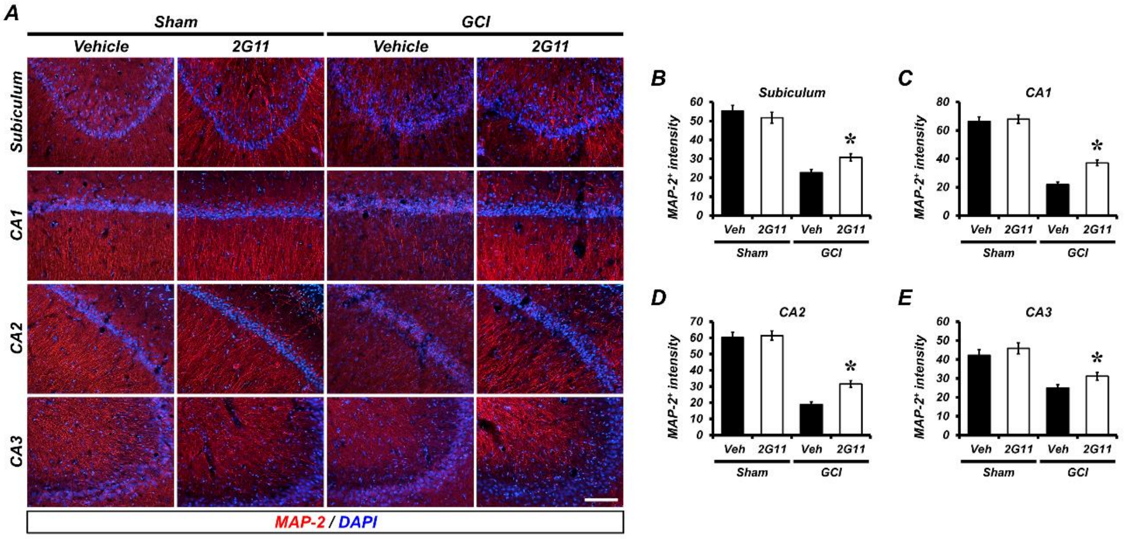

Figure S3. 2G11 treatment rescues GCI-induced microtubule damage. (**A**) Representative immunofluorescent images show the MAP-2 (red) with DAPI (blue) in the hippocampal subiculum, CA1, CA2 and CA3 region. Scale bar = 100 μm. (**B-E**) Quantification of the MAP-2 positive immunofluorescence intensity analyzed in hippocampal subiculum, CA1, CA2, and CA3 regions. Data are mean ± SEM; n = 5. $^*p < 0.05$ vs. the vehicle-treated GCI group (Kruskal–Wallis test followed by a Bonferroni post-hoc test; B: Chi square = 15.869, df = 3, $p = 0.001$; C: Chi square = 16.143, df = 3, $p = 0.001$; D: Chi square = 15.800, df = 3, $p = 0.001$; E: Chi square = 16.211, df = 3, $p = 0.001$).

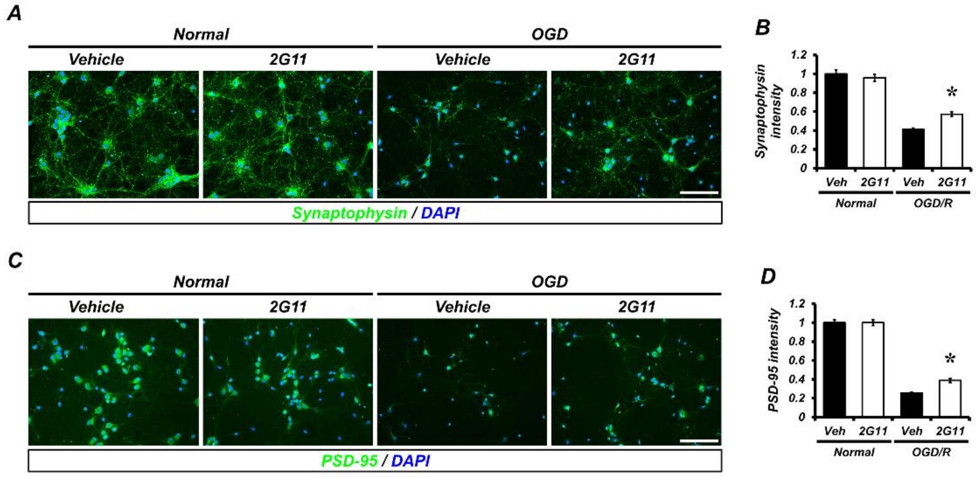

Figure S4. OGD/R-induced loss of synaptic proteins prevented by 2G11. (**A, C**) Representative images display the synaptic markers synaptophysin (**A**) and PSD-95 (**C**) in the vehicle and 2G11-treated cultured neurons. Scale bar = 100 μm. Immunoreactive intensity of synaptophysin (**B**) and PSD-95 (**D**) was analyzed with the mean gray value. Data are mean ± SEM; region of interest = 18-20. *$p < 0.05$ vs. vehicle-treated OGD/R group (Kruskal–Wallis test followed by a Bonferroni post-hoc test; B: Chi square = 58.002, df = 3, $p < 0.001$; D: Chi square = 56.729, df = 3, $p < 0.001$).

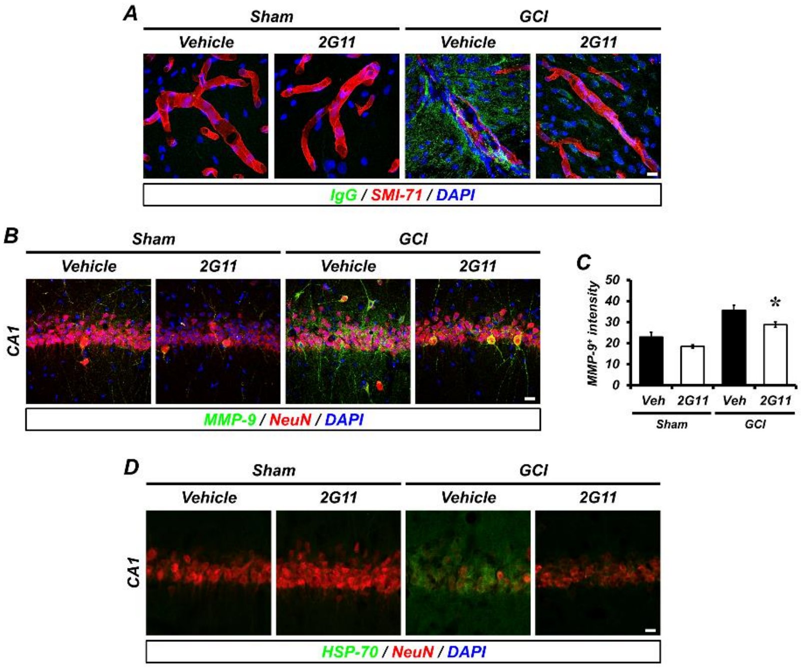

Figure S5. 2G11 treatment preserves BBB disruption and heat shock protein overexpression. (**A**) Double-label confocal micrographs of endogenous rat IgG molecules (green) and SMI-71^{+} endothelial cells (red) in the hippocampus from vehicle- and 2G11-treated rats after the sham surgery or GCI. Scale bar = 10 μm. (**B**) Double-label confocal micrographs of MMP-9 (green) and NeuN (red) in the hippocampal CA1 from vehicle- and 2G11-treated rats after the sham surgery or GCI. Nuclei are stained with DAPI (blue). Scale bar = 20 μm. (**C**) Bar graph showing the immunofluorescence intensity of MMP-9, as determined in the hippocampal CA1 region. Data are mean ± SEM; n = 6. *p < 0.05 vs. the vehicle-treated GCI group (Kruskal–Wallis test followed by a Bonferroni post-hoc test; Chi square = 11.667, df = 3, p = 0.009). (**D**) NeuN with HSP-70 double-labeled images in the hippocampal CA1.

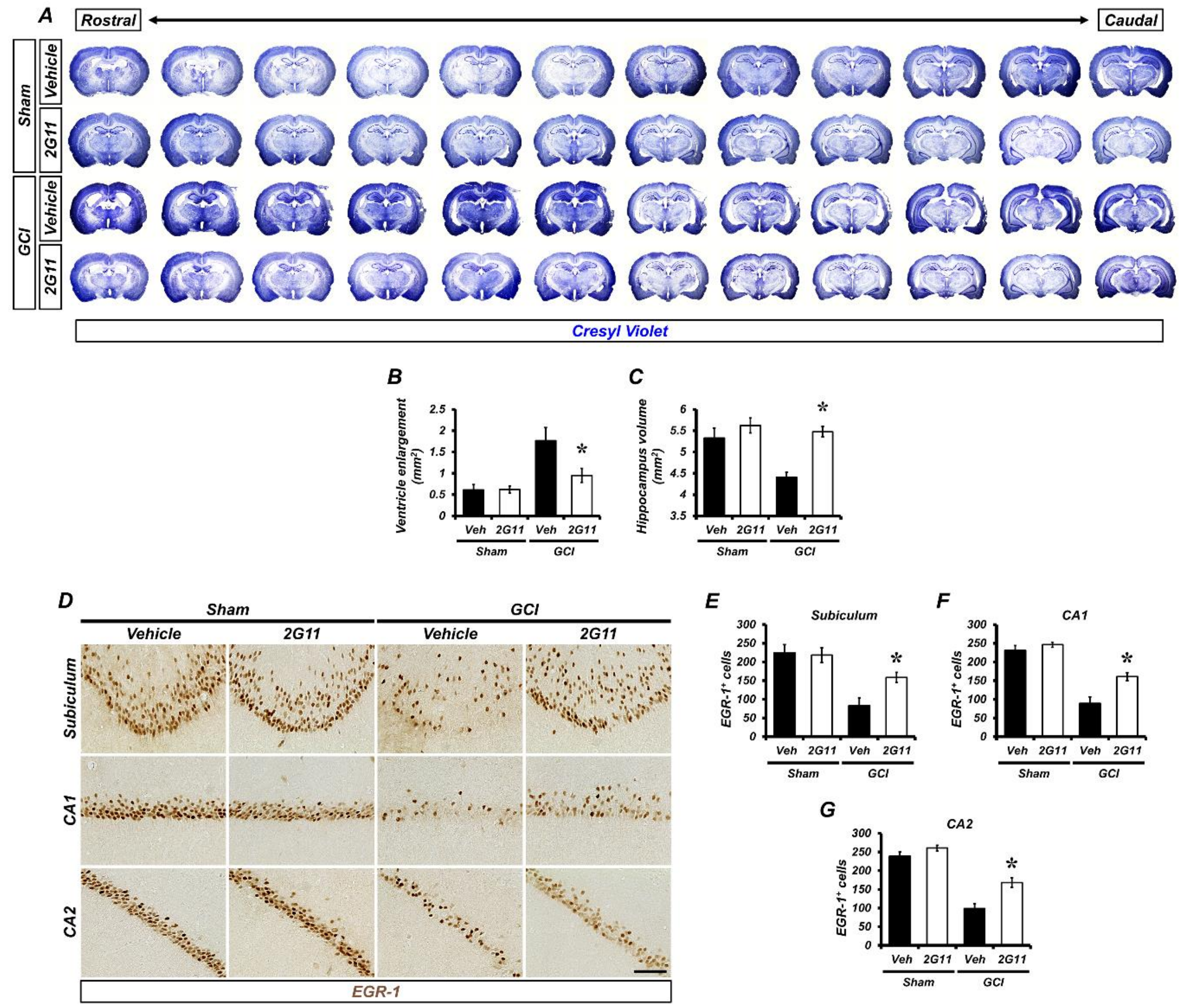

Figure S6. Chronic 2G11 treatment rescues GCI-induced brain atrophy, loss of synaptic plasticity and neuronal activity. (**A-C**) Cresyl violet staining and measurement of lateral ventricle and hippocampus volumes verify that 2G11 treatment rescues brain atrophy due to GCI. Data are mean ± SEM; n = 7-9 from each group. *$p < 0.05$ vs. the vehicle-treated GCI group (Kruskal–Wallis test followed by a Bonferroni post-hoc test; B: Chi square = 10.735, df = 3, $p = 0.013$; C: Chi square = 15.368, df = 3, $p = 0.002$). (**D**) Immuno-histological images show the EGR-1 positive cells in the hippocampal regions. Scale bar = 50 μm (**E-G**) Quantitative analysis of EGR-1 counts in hippocampal regions. Data are mean ± SEM; n = 6. *$p < 0.05$ vs. the vehicle-treated GCI group (Kruskal–Wallis test followed by a Bonferroni post-hoc test; H: Chi square = 13.812, df = 3, $p = 0.003$; I: Chi square = 18.037, df = 3, $p = 0.001$; J: Chi square = 18.098, df = 3, $p = 0.001$).

Table. S1 Modified neurological severity score points [1]

**Motor tests**	
Rasing rat by tail	3
Flexion of forelimb	1
Flexion of hindlimb	1
Head moved > 10° to vertical axis within 30s	1
Placing rat on floor (normal=0 ; maximum=3)	3
Normal walk	0
Inability to walk straight	1
Circling toward paretic side	2
Falls down to paretic side	3
**Sesonsory tests**	2
Placing test (visual and tactile test)	1
Proprioceptive test (deep sensation, pushing paw against table edge to stimulate limb muscles)	1
**Beam balance tests** (normal=0 ; maximum=6)	
Balances with steady posture	6
Graps side of beam	0
Hugs beam and 1 limb falls down from beam	1
Hugs beam and 2 limbs fall down from beam, or spins on beam (> 60s)	2
Attempts to balance on beam but falls off (> 40s)	3
Attempts to balance on beam but falls off (> 20s)	4
Falls off ; no attempt to balance or hang on to beam (< 20s)	5
**Reflex absence and abnormal movements**	6
Pinna reflex (head shake when auditory meatus is touched)	4
Corneal reflex (eye blink when cornea is lightly touched with cotton)	1
Startle reflex (motor response to a brief noise from snapping a clipboard paper)	1
Seizures, myoclonus, myodystony	1
**Maximum points**	18

Video. S1 Behaviors of vehicle-treated sham group. Beam balance tested by modified neurological severity score method.

Video. S2 Behaviors of 2G11-treated sham group. Beam balance tested by modified neurological severity score method.

Video. S3 Behaviors of vehicle-treated GCI group. Beam balance tested by modified neurological severity score method.

Video. S4 Behaviors of 2G11-treated GCI group. Beam balance tested by modified neurological severity score method.

References

1. Chen, J.; Sanberg, P.R.; Li, Y.; Wang, L.; Lu, M.; Willing, A.E.; Sanchez-Ramos, J.; Chopp, M. Intravenous administration of human umbilical cord blood reduces behavioral deficits after stroke in rats. *Stroke* **2001**, *32*, 2682-2688, doi:10.1161/hs1101.098367.
